# Association of rs142548867 (*EEFSEC*) and periodontitis Grade C in a young Brazilian population

**DOI:** 10.1590/1678-7757-2023-0058

**Published:** 2023-07-17

**Authors:** Camila Schmidt STOLF, Tiago TAIETE, Paloma A. do NASCIMENTO, Hélvis E. S. PAZ, Enílson Antônio SALLUM, Karina Gonzalez Silvério RUIZ, Márcio Zaffalon CASATI, Renato Corrêa Viana CASARIN

**Affiliations:** 1 Universidade Estadual de Campinas Faculdade de Odontologia de Piracicaba Departamento de Prótese e Periodontia Piracicaba SP Brasil Universidade Estadual de Campinas – UNICAMP, Faculdade de Odontologia de Piracicaba, Departamento de Prótese e Periodontia, Piracicaba, SP, Brasil.; 2 Universidade de Araras Departamento de Odontologia Araras SP Brasil Universidade de Araras, Departamento de Odontologia, Araras, SP, Brasil.; 3 Universidade Paulista Departamento de Periodontia São Paulo SP Brasil Universidade Paulista, Departamento de Periodontia, São Paulo, SP, Brasil.

**Keywords:** Genetics, Aggressive periodontitis, Whole Exome Sequencing, Genotype

## Abstract

Periodontitis Stage III-IV, Grade C (PerioC) is a severe form of Periodontitis. The individual genetic background has been shown to be an important etiopathogenic factor for the development of this disease in young, systemically healthy, and non-smokers patients. Recently, after exome sequencing of families with a history of the disease, PerioC was associated with three single nucleotide variations (SNVs) – rs142548867 (*EEFSEC*), rs574301770 (*ZNF136*), and rs72821893 (*KRT25*) – which were classified as deleterious or possibly harmful by prediction algorithms.

**Objective:**

Seeking to validate these findings in a cohort evaluation, this study aims to characterize the allele and genotypic frequency of the SNVs rs142548867, rs574301770, and rs72821893 in the Brazilian population with PerioC and who were periodontally healthy (PH).

**Methodology:**

Thus, epithelial oral cells from 200 PerioC and 196 PH patients were harvested at three distinct centers at the Brazilian Southern region, their DNA were extracted, and the SNVs rs142548867, rs574301770, rs72821893 were genotyped using 5′-nuclease allelic discrimination assay. Differences in allele and genotype frequencies were analyzed using Fisher’s Exact Test. Only the SNV rs142548867 (C > T) was associated with PerioC.

**Results:**

The CT genotype was detected more frequently in patients with PerioC when compared with PH subjects (6% and 0.5% respectively), being significantly associated with PerioC (odds ratio 11.76, p=0.02).

**Conclusion:**

rs142548867 represents a potential risk for the occurrence of this disease in the Brazilian population.

## Introduction

Periodontitis is an inflammatory disease of multifactorial etiology triggered by the host’s immune-inflammatory response to periodontopathogens present in the subgingival biofilm, clinically characterized by bone destruction and loss of attachment. In 2018, a world workshop classification indicated that the disease profile previously defined as Aggressive Periodontitis can now be defined as Periodontitis Stage 3-4, Grade C (PerioC) occurring in systemically healthy patients and non-smokers.^1,2^ This is a particularly severe form of the disease, with early onset, rapid progression, precocious edentulism, familial clustering of cases, and poor response to therapeutic approaches.^2-5^ This disease phenotype, although rare in developed countries, appears to be highly detected in undeveloped one, reaching around 5.5% of young population.^6^

The literature shows that individual genetic background is an important etiopathogenic factor that leads to this disease profile.^7-11^ Genome-wide association studies (GWAS), that is, the sequencing of the whole-genome or whole-exome of PerioC patients, have been performed to associate multiple genetic variants with the occurrence of PerioC.^12-14^ Although these studies came out with several associations between genes related to the immune response and this disease, none of them was performed within the Brazilian population. Knowing that ethnicity is an important factor that can change the individual genetic profile and that one of the most indicatives that this disease carries a genetic background is familial aggregation, a recent study of our research group conducted an analysis of the exome sequencing of families with a history of PerioC. The results showed that PerioC was associated with three single nucleotide variations – (SNVs), rs142548867, rs574301770 and rs72821893 – not described in the literature as associated with this disease. The three variants are classified as missense – when the exchange of a nitrogenous base results in the substitution of the encoded amino acid, which can generate structural and, consequently, functional alterations in the protein.^15^

The SNV rs142548867 is located at chromosome 3, 128264663 position, at eukaryotic elongation factor, selenocysteine-tRNA specific (*EEFSEC)* gene, encoding eEFSec protein, and is responsible for the inversion of the nitrogenous base C for T (c.668C>T). This variation results in the replacement of the amino acid Proline (Pro) by a Leucine (Leu) residue at protein position 223 (p.Pro223Leu). This is an important translation factor for selenoproteins and selenoenzymes, which are critical for maintaining the tissue homeostasis, and regulation of immune-inflammatory cells.^16,17^ Rs574301770 (c.466C>G) is located on zinc finger protein 136 (*ZNF136*) gene, 12186844 position on chromosome 19, being responsible for the inversion of the amino acid Arginine for a Glycine at position 156 (p.Arg156Gly) at zinc finger protein 136. This gene presents a DNA-binding transcription factor activity, RNA polymerase II-specific, being involved in transcriptional regulation. The last variation reported on the previous study, rs72821893 (c.800G>A), is located at 40751196 position on chromosome 17, on Keratin 25 (*KRT25*) gene. It results in the change of the amino acid Arginine for a Histidine, at 267 location (p.Arg267His) of Keratin, Type I cytoskeletal 25 protein. This protein exhibits an essential role on the proper assembly of types I and II keratin complexes and cytoskeletal organization.

As the three variants found were classified as deleterious by the SIFT prediction algorithm – and the variations in the *EEFSEC* and *ZNF136* genes were predicted as possibly harmful to protein synthesis by the Polyphen algorithm^15^ – this study proposed to evaluate the allele and genotypic frequency of the SNVs rs142548867, rs574301770, and rs72821893 in a Brazilian population with PerioC and a control Brazilian population who were periodontally healthy (PH).

## Methodology

### DNA obtention

#### Patients eligibility criteria

For the elaboration of this study, the STREGA checklist has been followed by the authors.^18^ All the DNA samples used in this study were first collected in a previous study,^19^ carried out between 2016 and 2018 at a Brazilian University, in which 196 periodontal healthy (PH) and 200 PerioC (previously as aggressive periodontitis) patients were selected. After the approval of Ethics Committee (number 58679416.4.0000.5418), all patients signed an informed consent form to participate in this research.

All the patients recruited were identified based on the 1999 International Workshop for the Classification of Periodontal Diseases and Conditions.^2^ To provide the correct patient’s diagnosis, the following clinical parameters were assessed in all patients: full-mouth plaque index,^20^ full-mouth bleeding on probing score,^21^ and Probing Pocket Depth (PPD) at six points around each tooth.

For inclusion, Perio4C patients needed to be, at the time of diagnosis, under 35 years old – to characterize the rapid progression disease – present at least 20 teeth, and at least 8 teeth with PPD≥5 mm, with bleeding on probing (having at least 2 with PPD≥7 mm).^22^ These criteria also correspond to a Periodontitis Stage 3-4, Grade C, generalized according to the recent Classification.^1^ PH individuals should not have interproximal attachment loss and PPD>4 mm.^22,23^ Since age does not interfere with the genetic background of the patient, no criteria were stablished for the control group.

All the patients from both groups were recruited from three distinct Universities from South Brazil, including the Research Center where the analysis of this study was carried out. PerioC patients who were seeking treatment or were enrolled in Supportive Periodontal Therapy (SPT) at the Dental School’s centers were included. A calibrated team at each center evaluated the clinical and demographical data, as well as performed the patient treatment. PH subjects were included from patients who sought other treatments at the centers (e.g., aesthetic, restorative, and prosthetic) and who, after periodontal examination, were found not to have the disease.

Exclusion criteria adopted for all patients were smoking or previous history of smoking, diabetes, hepatitis, other systemic conditions (e.g., cardiovascular disease, HIV, etc.), use of immunosuppressive drugs, prolonged use of anti-inflammatory medications, use of orthodontic device, and diseases of the oral hard and soft tissues (except caries and periodontitis).^24^

#### Cell collection and DNA extraction

The method used to do the genomic DNA isolation from epithelial oral cells are fully described by Trevilatto and Line^25^ (2000). Briefly, the patients undertook a mouthwash containing 5 mL 3% dextrose solution for 60 seconds. Three mL of TNE solution (17 mM Tris/HCl [pH 8.0], 50 mM NaCl, and 7 mM EDTA) in 66% ethanol was added to the sample tube collection. Samples were centrifuged (3000 rpm for 10 min), the supernatant was discarded, and the pellet resuspended in 500 μL of extraction buffer (10 mM Tris-HCl [pH 8.0], 5 mM EDTA, 0.5% SDS) and 10 μL of proteinase K (Sigma Chemical Co., St. Louis, MO) (20 mg/ml) was added. DNA was dissolved in nuclease-free water, and its quantity was evaluated spectrophotometrically using a spectrophotometer (Nanodrop 2000 device, Thermo Scientific, Wilmington, DE).

## SNVs detections

This study was conducted based on previous results obtained by the research group, where the SNVs rs142548867 (*EEFSEC*), rs574301770 (*ZNF136*), rs72821893 (*KRT25*), and the indel rs371464745 (*GPR6CA*) were identified as closely related to PerioC development.^15^

For the detection of rs142548867 (*EEFSEC*), rs574301770 (*ZNF136*), and rs72821893 (*KRT25*) (Table 1) a PCR-based genotyping was performed on the thermocycler (LightCycler 480, Roche Diagnostics, GmbH, GR) using predesigned 5’-nuclease allelic discrimination assays (TaqMan – Thermo Scientific). PCR were carried out in a total of 10 μl, containing 20 ng of gDNA, 5 μl of a genotyping master mix (Thermo Scientific), 0.5 μl probes assay mix 20 × (Thermo Scientific). The reactions were performed under the following conditions: 10 minutes at 95°C, 40 × (15 seconds at 95°C, 1 minute at 60°C), as recommended by the manufacturer. The frequency of failure at the genotyping process was 3.6% for rs72821893 (*KRT25*), 5.9% for rs142548867 (*EEFSEC*), and 2.1% for rs574301770 (*ZNF136*). After retesting the samples that failed at first, this frequency dropped to around 0.5% per single nucleotide polymorphism tested. After the genotyping, reactions were randomly repeated in 10% of the samples for each SNV for quality control purposes, and the concordance rate was 100%.^26^

**Table 1:** rs142548867 (EEFSEC), rs574301770 (ZNF136), and rs72821893 (KRT25) characteristics

Existing Variation	Position	Gene / Protein	NCBI (Gene / Protein)	SNVs and Aminoacid Change	PolyPhen prediction (score)	SIFT prediction (score)	Consequence
rs142548867	3:128264663-128264663	*EEFSEC* (eukaryotic elongation factor, selenocysteine-tRNA specific)	NM_021937.4 / NP_068756.2	c.668C>T (p.Pro223Leu)	Possibly damaging (0.843)	Deleterius (0.01)	Missense
rs574301770	19:12186844-12186844	*ZNF136* (zinc finger protein 136)	NM_003437.3 / NP_003428.1	c.466C>G (p.Arg156Gly)	Probably damaging (0.935)	Deleterius (0.03)	Missense
rs72821893	17:40751196-40751196	*KRT25* (keratin 25)	NM_181534.3 / NP_853512.1	c.800G>A (p.Arg267His)	Benign (0.329)	Deleterius (0.02)	Missense

## Statistical Analysis

First, the Shapiro-Wilk test was used to access the normality of all the data obtained. For clinical and demographical data statistical evaluation were used Student’s t-test and Chi-square (for gender). The distribution of genotypes for each SNV was evaluated for deviation from Hardy-Weinberg Equilibrium and differences in genotypic and allelic frequencies of each SNV between groups were evaluated by Fisher’s Exact Test (BioEstat 5.0 Software). A significance level of 5% was considered to be statistically significant.

Power calculations were performed using a specific software (Quanto 1.2.4 software, University of Southern California, CA), assuming a prevalence of PerioC in Brazil of 0.0549,^6^ 1:1 case/control ratio, the SNV’s previously reported (1000 Genomes Study) minimum allele frequencies (MAFs), and a 5% error rate in an allelic test.

## Results

### Study Population

Table 2 shows the clinical and demographical data of the study participants. Regarding demographical data, age, gender (male and female proportions), and Ethnicity (European and African ancestry) were considered. Both age and gender presented statistical difference between PerioC and PH groups (p<0.0001 and 0.001, respectively). Skin color evaluation showed that 83.5% and 86.2% of our sample with PerioC and PH, respectively, presented European characteristics, against 16.5% and 13.8% of African ancestrally, being similar between groups with no statistical difference (p=0.845) and showing that a mixed cohort was included, representing the Brazilian population.^27^

**Table 2:** Clinical and demographical data of the study participants

Characteristics	PerioC	PH	p-value
Age	34.0±4.6	30.5±5.8*	<0.0001
Gender (n Male (%))	42 (21)	69 (35)^†^	0.001
Ethnicity (n European (%))	167 (83.5)	169 (86.2)	0.485
Plaque Index	23.1±6.5	19.1±5.4*	<0.0001
Bleeding on Probing	24.8±9.0	19.2±2.3*	<0.001
Probing Pocket Depth (PPD)	2.35±0.0	2.1±0.2	>0.05
PPD ≥ 5 mmt (n)	41.32±12.1	0	-

* Indicate statistical difference (Student’s t-test, p < 0.05). ^†^ Indicate statistical difference (Chi-Square test, p < 0.05).

Age, Plaque Index, Bleeding on Probing, Probing Pocket Depth (PPD) and PPD ≥ 5 mm are represented by mean ± standard deviation. Age: evaluation of male and female proportions. Ethnicity: evaluation of Caucasian and African ancestral proportions. t PPD ≥ 5 mm: Sites presenting a probing pocket depth ≥ 5 mm at diagnosis.

The evaluation of the clinical parameters showed that both Plaque Index and Bleeding on Probing presented statistical difference between diseased and healthy groups (p<0.0001 and <0.001, respectively). There were no statistical differences observed at PPD between PerioC and PH groups (p>0.05).

### Genotyping and Power Analysis

The genotype frequencies observed for the three SNVs evaluated in PH and PerioC groups were not statistically different from those expected on the Hardy-Weinberg equilibrium. The only SNV that could be associated to PerioC with statistical significance in the Brazilian cohort was the missense SNV rs142548867 (C>T) at *EEFSEC* gene. For PerioC the allele frequencies were 192 and 96% for C allele and 4% for the rare T, and regarding the genotype, a frequency of 93%, 6%, and 1% were detected for CC, CT, and TT. In PH group, the frequencies of CC, CT, and TT were 99%, 0.5%, and 0.5%, respectively, resulting in a C allele frequency of 99% and 1% of T. The rare allele was detected more frequently in patients with PerioC when compared with PH subjects (4% and 1%, respectively), and, consequently, the CT genotype was more present at the affected population compared with the healthy one (6% and 0.5%, respectively), being significantly associated with PerioC (odds ratio 11.76, p=0.02), making this SNV a potential risk for the occurrence of this disease. Table 3 shows the results of allele and genotype frequencies.

**Table 3:** Allele and Genotype frequency of rs142548867, rs574301770, and rs72821893 SNVs

SNV		PerioC (n (%)	PH (n (%)	Reason of Possibilities PerioC x PH (95% CI) p-valor
**rs142548867 (*EEFSEC*)**				
HWE p-valor		0.83	0.52	p<0.05
Allele (C / T)	C	192 (96)	194 (99)	p=0.056
	T	8 (4)	2 (1)	
Genotype (CC / CT / TT)	CC	186 (93)	194 (99)	Reference
	CT	12 (6)	1 (0.5)*	11.76 (1.5-89.6) p=0.02
	TT	2 (1)	1 (0.5)	p=0.51
**rs574301770 (*ZNF136*)**				
HWE p-valor		0.94	0.7	p<0.05
Allele (C / G)	C	193 (96.5)	192 (98)	p=0.28
	G	7 (3.5)	4 (2)	
Genotype (CC / CG / GG)	CC	188 (94)	190 (97)	Reference
	CG	10 (5)	4 (2)	p=0.09
	GG	2 (1)	2 (1)	p=0.68
**rs72821893 (*KRT25*)**				
HWE p-valor		0.68	0.87	p<0.05
Allele (G / A)	G	198 (99)	195 (99.5)	p=0.51
	A	2 (1)	1 (0.5)	
Genotype (GG / GA / AA)	GG	199 (98)	195 (99)	Reference
	GA	1 (2)	1 (0.5)	p=0.75
	AA	0	0	p=1.0

* Indicate statistical difference (Fisher’s Exact Test, p < 0.05).

Regarding the other two SNVs, rs574301770 and rs72821893, no statistical difference could be detected when comparing the affected and the control group. rs574301770 (C>G) presented an allele frequency of 3.5% for G allele, and 96.5% for C at PerioC group and 2% and 98% of C and G alleles for PH. rs72821893 (G>A) had an allele frequency of 1% for the rare allele and 99% for the ancestral one for PerioC population and 0.5% and 99.5% for PH.

Power analysis showed optimal statistical power (80% or more) to detect associations with the current sample size for the variations rs142548867 (94.1%) and rs574301770 (99.73%) when comparing PerioC and PH populations. For the same comparation, the rs72821893 SNV showed a statistical power of 79.5%.

## Discussion

In this study, the missense SNV rs142548867 (C > T), in *EEFSEC* gene, was associated with PerioC, since the rare T allele was found more frequently in this population than in PH. This higher frequency, in turn, leads to a higher frequency of the heterozygous genotype (CT) when compared with PH group – 6% and 0.65% respectively, resulting in an odds ratio of 11.76 for PerioC occurrence (p=0.02). Meanwhile, SNVs rs574301770 (*ZNF136*) and rs72821893 (*KRT25*) weren’t associated to PerioC (p>0.05). It is important to notice that this research was conducted based on the previous results of our group. In that study, a familial whole-exome of PerioC patients – two affected probands, their parents (one with and one without a history of this disease), and one sibling (healthy patient) – were carried out, searching for genetic risk factors related to this disease. The research group identified, besides that the healthy family members have high genetic similarity (though it can be used to exclude benign variations when compared to the genetic profile of the affected patient), that the following SNVs are closely related to PerioC development: rs142548867 (*EEFSEC*), rs574301770 (*ZNF136*), and rs72821893 (*KRT25*), and also indels in GPRC6A and ELN: In silico analysis indicated a functional impact of the indel rs371464745 in GPR6CA. Further, unrelated patients’ genotyping confirmed its association with PerioC. However, up to date, there was no study confirming the missense variations occurrence to PerioC in a populational-based study.

The disease-correlated SNV is located at chromosome 3, 128264663 position, at *EEFSEC* gene (encodes eEFSec protein), and is responsible for the inversion of the nitrogenous base C for T. This variant is classified as a missense one, that is, the substitution of the base pair can result in the replacement of one amino acid (protein building block) to another during the translation process. In this case, this base pair inversion results in the replacement of the amino acid Proline (Pro) by a Leucine (Leu) residue at protein position 223 (p.Pro223Leu). Thus, the inclusion of the Leucin on eEFSec protein instead of Proline is involved on a higher risk of developing PerioC. Structural and in silico analysis showed that this substitution was not predicted to promote conformational changes in the eEFSec protein structure.^15^ Nevertheless, PolyPhen-2 prediction program pointed to a probability of 0.843 for this SNV to be a possible damaging substitution. PolyPhen-2 is a software that has been used to predict and estimate the probability of the missense mutation being damaging, based on functional annotation of variants, transcripts, and protein sequences, building conservation profiles.^28^

*EEFSEC* gene, eukaryotic elongation factor selenocysteine-tRNA specific, is an important translation factor for selenoproteins and selenoenzymes, which are critical for maintaining redox potential – maintaining non-damage tissue levels of reactive oxygen species (ROS) – tissue homeostasis, and regulation of immune-inflammatory cells.^16,17^ *EEFSEC* has never been directly associated with PerioC or other periodontitis phenotypes before.^16,17,29^ Meanwhile, oxidative stress has been closely related to PerioC occurrence, as high levels of oxidative stress markers – as ROS – are related to this phenotype most than other diseases. Meaningfully, an increase in ROS activity can impact tissue homeostasis, immune-inflammatory cascade, and this disease pathogenesis.^30^

The human body produces ROS, highly reactive derivatives of the oxygen metabolism, as a consequence of phagocytic infiltration when the host defense against bacterial pathogens is activated.^7,8,31^ Although they are important in several cellular processes, ROS are also known to be potentially damaging to cells, and consequently tissues, since it triggers transcription events, leading to uncontrolled expression of lipids and proteins when there is a failure in our body’s antioxidant systems to neutralize it or when its production is increased, leading to oxidative stress.^32-34^ Based on the above and since this study showed an association between the SNV observed in the *EEFSEC* gene and the occurrence of PerioC, it can be assumed that this may act adversely on the synthesis of this protein, causing a decrease or impairment in its synthesis process, altering the oxidative stress control of immune-inflammatory cells, which in turn would affect the inflammatory response in patients with PerioC.^16^

Regarding the crosslink between ROS and PerioC, D’Aiuto, et al.^33^ (2010) evaluated the blood serum of 145 PerioC patients and 56 health controls after 1, 3, 5, 7, and 30 days of patient’s treatment (first intervention) regarding the production of Diacron-reactive oxygen metabolites (D-ROM), antioxidant potential, C-reactive protein (CRP), interleukin-6, and lipid profiles. Patients with severe periodontitis exhibited higher D-ROM levels and lower total antioxidant capacity (p<0.001) when compared with control group, associating the PerioC disease with oxidative stress.^33^ Singer, et al.^34^ (2009) also associated systemic oxidative stress with PerioC immune response. They examined the relationship between serum levels of 8-isoprostane (reference molecule for increased systemic oxidative stress) and IgG antibodies against 17 microorganisms in oral biofilm, in which 8-isoprostane was associated with the extent of periodontal disease severity, and the total IgG antibody directed to the oral biofilm was significantly associated with periodontal disease severity, plaque, and serum 8-isoprostane (p<0.0001). The authors concluded that increased systemic oxidative stress is associated with a generalized decrease of serum IgG antibody responses to the oral biofilm.^34^

Secondary damage also can be correlated with rs142548867 at *EEFSEC*. Taiete, et al.^15^ (2020) also demonstrated, by Protein-Protein Interaction (PPI) network analysis, that both this SNV and the ones at *ZNF136* (rs574301770) and *KRT25* (rs72821893) were linked to one same major node – ubiquitin C (UBC) among PerioC patients,^15^ in other words, by affecting the production of eEFSec protein this SNV could also affect Ubiquitin C production by an alteration at its synthesis pathway. The proteins encoded by UBC gene are mainly responsible for regulating transduction signal and protein degradation, playing an important part in regulating the innate and adaptive host immune responses.^35,36^ This way, its entirety is well-established by biological processes involved in periodontitis pathogenesis, like the MAPK pathway, antigen processing and presentation via MHC-I, and apoptotic processes, being reported as an important gene for periodontitis in an interactome study.^37^

Currently, no association between SNVs rs574301770 and rs142548867 and other inflammatory diseases has been reported in the literature. However, the SNV rs72821893 (*KRT25)* was recently associated with the individual’s response to asthma treatment.^29^ Leusink, et al.^29^ (2016), when studying a population of 110 children with asthma diagnosis that was not well controlled despite inhaled corticosteroid (ICS) treatment and the 17q12-21 locus – previously associated with childhood asthma – found that the rs72821893 in *KRT25* gene was associated with the lung function and airway hyperresponsiveness (AHR) to methacholine, and additionally with ICS treatment response.^29^

Regarding the allelic frequency of the SNVs studied, the PH population (Brazilian), on this study, reported an ancestral allele frequency of 99% for rs142548867 (C>T), of 98% for rs574301770 (C>G), and of 99.5% for rs72821893 (G>A), resulting in a very low frequency of the altered allele in all three variations studied. The Global MAF reported for these SNVs at the 1000 Genomes study shows a very similar frequency for ancestral allele: 99.8% for rs142548867 (C>T), 99.9% for rs574301770 (C>G), and 99.4% for rs72821893 (G>A).^38^ Although a low frequency of the altered allele can be observed for all three SNVs, a difference between CT genotype of PerioC and PH groups could be detected for rs142548867 (p=0.028).

Recent studies have shown that, in certain cases, the control group used to evaluate genetic susceptibility to periodontal disease may be biased, considering that we can have patients carrying the same SNVs associated with the disease but do not develop it because of their good oral hygiene.^39,40^ In this study, we evaluated three SNVs that can be associated with PerioC, a disease that mostly affects young patients, around 20-30 years old or even younger.^6^ In this case, establishing a control group is always challenging because, at this similar age, we are not certain that the periodontally healthy patients selected to compose the control group will not develop the disease in the future. On the other hand, we choose a control group with similar ages because, in young people, environmental or local etiological factors of periodontal disease, like poor oral hygiene, do not have enough impact on initiating an aggressive disease, such as PerioC or do not have sufficient time to develop a chronic disease, such as Grade A or B Periodontitis. Furthermore, PerioC is a multifactor disease, and although dental plaque continues to be the primary etiological factor for developing this disease, it does not have much effect on the aggressiveness of PerioC. In fact, one of the characteristics of the rapid progression periodontitis is the presence of massive alveolar bone loss incompatible with the biofilm deposits present in these patients, who also have a poor response to periodontal treatment and bacterial control therapies.

Another aspect of the demographic characteristics of the cohort included in this study is the ancestrally of the Brazilian population. GWAS have recently demonstrated that it is important to validate genetic polymorphisms, or variations, in individual populations due to the unique genetic background of each country or region. In this context, the Brazilian population is highly miscegenated, with a large number of people with African and European ancestrally, despite, sometimes, not showing any clear physical characteristics of these ethnicities^27^; Thus, this high miscegenation makes the Brazilian population difficult to segregate according to the “ancestry,” but this also makes this population unique.^41,42^ In our study, we included a mixed cohort, with 16.5% declaring to be African descendants and 83.5% European for PerioC subjects, representing the percentage of Brazilians with European and African ancestry for the studied region.

Although our study showed an association between the SNV missense rs142548867 and PerioC, with probable negative repercussions in the synthesis of the eEFSec protein, future studies are necessary to explain the molecular mechanisms behind the lack of this molecule and the occurrence of PerioC, either this is associated with an increase in ROS levels in these patients or not, as well as the validation of this association in independent populations other than the Brazilian one. Furthermore, it can be assumed that the SNV rs72821893 was not associated with PerioC, probably due to the MAF observed for Brazilians (around 0.6%). This low frequency calls for a greater number of volunteers to obtain adequate statistical power (80% or more) to refute or not the previously reported association. Despite the need for future studies, this line of study has brought significant results that may help researchers to elucidate the genetic character behind the etiopathogenesis of PerioC.

## Conclusion

It can be concluded that the SNV rs142548867 in the *EEFSEC* gene was associated with PerioC within the studied population, and that this missense variation presents itself as a risk indicator for the occurrence of PerioC.
